# The role of CD133 in cancer: a concise review

**DOI:** 10.1186/s40169-018-0198-1

**Published:** 2018-07-09

**Authors:** Paige M. Glumac, Aaron M. LeBeau

**Affiliations:** Department of Pharmacology, University of Minnesota Medical School, Nils Hasselmo Hall 3-104, 312 Church St. SE, Minneapolis, MN 55455 USA

**Keywords:** Cancer stem cells, CD133, Cancer, Prognosis, Immunotherapeutic

## Abstract

Despite the abundant ongoing research efforts, cancer remains one of the most challenging diseases to treat globally. Due to the heterogenous nature of cancer, one of the major clinical challenges in therapeutic development is the cancer’s ability to develop resistance. It has been hypothesized that cancer stem cells are the cause for this resistance, and targeting them will lead to tumor regression. A pentaspan transmembrane glycoprotein, CD133 has been suggested to mark cancer stem cells in various tumor types, however, the accuracy of CD133 as a cancer stem cell biomarker has been highly controversial. There are numerous speculations for this, including differences in cell culture conditions, poor in vivo assays, and the inability of current antibodies to detect CD133 variants and deglycosylated epitopes. This review summarizes the most recent and relevant research regarding the controversies surrounding CD133 as a normal stem cell and cancer stem cell biomarker. Additionally, it aims to establish the overall clinical significance of CD133 in cancer. Recent clinical studies have shown that high expression of CD133 in tumors has been indicated as a prognostic marker of disease progression. As such, a spectrum of immunotherapeutic strategies have been developed to target these CD133^pos^ cells with the goal of translation into the clinic. This review compiles the current therapeutic strategies targeting CD133 and discusses their prognostic potential in various cancer subtypes.

## Background

Cancer is the second leading cause of death in the United States and a major cause of mortality and morbidity worldwide [1, 2]. Despite the social and economic impact of cancer on society, it has been exceedingly difficult to treat even the most common malignancies due to the heterogeneous nature of the disease [3]. The tumor mass consists of heterogeneous cell populations that are affected intrinsically by genetic and epigenetic alterations and extrinsically by the host microenvironment [4–6]. Until recently, the most common approach towards cancer treatment has largely focused on targeting tumor progression based on the clonal evolution model, which hypothesizes that the vast majority of cancer cells have the ability to proliferate, self-renew, drive tumor growth, initiate metastasis, and develop therapeutic resistance [3]. This stochastic model posits that most malignancies arise from a single clone which becomes genetically unstable and selective pressure from the host microenvironment facilitates the growth and survival of this subpopulation resulting in intratumoral heterogeneity [7–9]. While the clonal evolution model has been clearly described as the basis for tumor progression in various cancer subtypes [10–17], treatment strategies which target the bulk of the tumor cells have been relatively limited due to cancer recurrence [3].

Several studies have suggested that the cancer stem cell (CSC) hypothesis may be a more accurate model for describing tumor development, progression, and recurrence post-treatment. The CSC hypothesis follows a hierarchical model in which only a small subset of the cells within the tumor are able to self-renew, differentiate, and ultimately drive tumor growth [5, 18]. Since CSCs possess multilineage differentiation potential, they are thought to be the driving factor for intratumoral heterogeneity, cancer metastasis and radio/chemotherapeutic resistance [19–22]. To better understand the molecular basis through which CSCs promote tumor progression, metastasis, and therapeutic resistance, numerous studies have identified biomarkers on the surface of CSC populations to distinguish them from the bulk of the tumor cells. CD133 (also known as AC133 and prominin-1) is the most frequently used cell surface antigen to detect and isolate CSCs from various solid tumors [23], including brain, colon, pancreas, prostate, lung, and liver. There has recently been, however, some contrasting evidence of the accuracy associated with using CD133 as a marker for CSC detection and/or isolation. This review aims to discuss the clinical relevance of CD133 in cancer and thoroughly describe the utility and limitations of using CD133 for CSC identification and therapeutic targeting.

## Structure and function of CD133

**Fig. 1:**
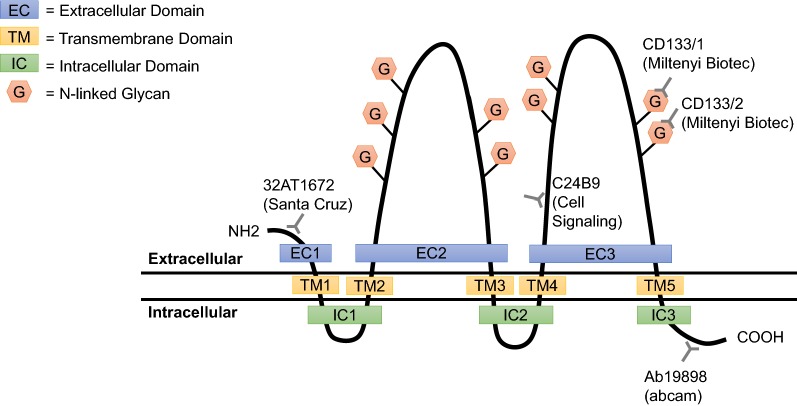
Schematic of the CD133 topology and putative epitopes of commercially available CD133 antibodies. The five transmembrane glycoprotein contains two large extracellular loops (EC2 and EC3), which comprise a total of nine N-linked glycan residues. The commonly used CD133/1 and CD133/2 epitopes are located on the EC3 region of CD133 and have the potential for epitope masking or antibody inaccessibility due to changes in glycosylation patterns

CD133 is a 97 kDa pentaspan transmembrane glycoprotein that contains an extracellular N-terminal domain (EC1), five transmembrane segments which separate two small intracellular loops (IC1 and IC2), two large extracellular loops (EC2 and EC3), and an intracellular C-terminal domain (IC3) [24] (Fig. 1). The two extracellular loops contain nine putative N-glycosylation sites; five on EC2 domain and four on EC3 domain [25]. Glycosylation of CD133 yields a 120 kDa protein and alters the overall tertiary structure and stability of CD133 [26–28]. The CD133 gene, prominin 1 (*PROM1*), is located on chromosome 4 in humans and chromosome 5 in mice and is only approximately 60% homologous from primates to rodents [28, 29]. Transcription of human CD133 is driven by five alternative promoters, three of which are located on CpG islands and are partially regulated by methylation. These promoter regions often result in alternative splicing of CD133 mRNA, resulting in CD133 structural variants with potentially unique roles [27, 30–32].

The physiologic function of CD133 in normal biology and the progression of cancer remains elusive. CD133 is known to be preferentially localized in plasma membrane protrusions and microvilli, suggesting its involvement in membrane organization [33, 34]. The subcellular localization of CD133 allows it to bind directly to cholesterol-containing lipid rafts where it can be involved in various signaling cascades [35]. Observations from CD133 knockout mice support the presumed role of CD133 as a scaffolding protein by showing that a lack of CD133 caused a defect in outer segment morphogenesis of the photoreceptor cells. While these mice remained viable and fertile, they experienced significant retinal degeneration and blindness [36]. Other studies have additionally suggested a potential role of CD133 in determining cellular fate or maintaining stem cell-like properties [37–40], however, the precise molecular mechanisms for this are still unclear.

Many different molecular mechanisms have been investigated to better understand the modulation of CD133 in normal and cancer stem cells. Studies from both normal and cancer stem cell lines have indicated that CD133 antibody reactivity is reduced when cells are in the G_1_/G_0_ portion of the cell cycle as opposed to the G_2_/M phase of the cell cycle, suggesting some level of cell cycle dependence related to CD133 expression [41]. Hypoxia in the stem cell and tumor microenvironment has also been shown to promote CD133 expression via hypoxia inducible factor-1α (HIF-1α) upregulation [42–45]. Similarly, a study using human glioma cells demonstrated that pharmacologically induced mitochondrial dysfunction produced an increase in CD133 protein expression, suggesting that hypoxia may also be perturbing the mitochondrial membrane potential to regulate CD133 post-transcriptionally [46]. It has also been suggested that CD133 may play an important role in cellular glucose metabolism through modulation of the cytoskeleton [47]. In parallel to these roles, a study by Bourseau-Guilmain discovered a mechanism by which CD133 inhibited transferrin uptake [48]. Since transferrin is involved in supplying iron to the cell and iron is required for efficient oxygen transport, the CD133-transferrin-iron network may provide a potential mechanism for a better understanding of CD133 modulation under hypoxic conditions.

Several reports have also begun to highlight potential signaling pathways involved in CD133 expression. The role of CD133 as an inductor of Wnt/β-catenin signaling has been previously reported in CSCs [49–51]. In particular, suppression of CD133 was associated with a loss of β-catenin nuclear localization and a reduction in canonical Wnt signaling [49, 50]. Similar results were also reported in normal CD133^pos^ renal cells, suggesting that CD133 may be a functional protein and/or a marker of differentiation status [52]. Additionally, the deacetylase, HDAC6, has been shown to physically interact with CD133 in mammalian cells [51]. This association stabilized β-catenin, whereas inhibition of either CD133 or HDAC6 resulted in increased β-catenin acetylation and degradation and correlated with decreased proliferation and tumorigenesis, suggesting a potential target for cancer therapy. CD133 has also been implicated as an important regulator of PI3K/Akt signaling in CSCs [53–55], however, due to the complexity of the biological role of CD133, most studies focus on its use as a cell surface marker for the detection of somatic stem cells and CSCs. The functional role of CD133 is even less clear in the context of cancer, as it is ubiquitously expressed in numerous malignant and non-malignant tissues [56].

## CD133 as a stem cell marker

CD133 alone, or in combination with other markers, has recently been used to identify stem cells from a variety of tissues. To determine the validity of CD133 as a marker for somatic stem cells in a tissue, however, one must first understand the biology of the cells expressing the CD133 and the distribution pattern of the cells within each tissue type.

### CD133 in hematopoietic stem cells

CD133 was initially discovered as a hematopoietic stem cell (HSC) marker in 1997 [57, 58]. In human HSCs, the biological function of CD133 has been linked to stem cell-fate decisions and emerges as an important physiological regulator of stem cell maintenance and expansion [59]. A recent study showed that CD133 was expressed by murine HSCs, however, it appeared to play an insignificant role in HSC function during steady-state and stress-induced hematopoiesis. This study also showed that CD133 was important for the normal recovery of red blood cells during myelotoxic stress, such as in the case of chemotherapy treatment. These data suggest that while CD133 is likely not a critical regulator of early HSC function, it may play a role in early myeloerythroid function during stress hematopoiesis [60]. The lack of functional consequences on murine hematopoiesis in the absence of CD133 may represent a stark species difference between mice and humans, considering several other studies have indicated that CD133 is a critical regulator of HSC differentiation and function in humans [38, 59].

### CD133 in neural stem cells

CD133 has been used as a marker to identify and isolate neural stem cells (NSCs). Primary human central nervous system stem cells have been derived from fresh human fetal brain tissue using fluorescence-activated cell sorting (FACS) and the monoclonal antibody (mAb), 5F3, which recognizes CD133. Sorted cells expressing CD133^pos^/CD34^neg^/CD45^neg^ phenotype initiated neurosphere cultures, which exhibited self-renewal and differentiation potential. Additionally, these CD133^pos^ cells showed potent engraftment, proliferation, migration, and neural differentiation upon transplantation into the brains of NOD-SCID newborn mice [61, 62]. Similarly, CD133 has also been used to isolate NSCs from the cerebellum of mice [63]. A more recent study using cultured NSCs demonstrated that while CD133 is expressed heterogeneously in undifferentiated human NSC cultures, stem cell potency is not exclusive to CD133^pos^ populations. In fact, clonogenicity was significantly higher in CD133^neg^ cell populations. These cells were disproportionately represented in G0/G1 cell cycle phase, while CD133^pos^ NSCs resided predominantly in the S, G2, or M phases, supporting the previously mentioned notion that CD133 expression may be partially cell cycle dependent [64]. This suggests that CD133 may provide a distinction between proliferative and quiescent cells and thus should be used cautiously as a putative marker of a stable, distinct stem cell population.

### CD133 in prostate stem cells

**Fig. 2:**
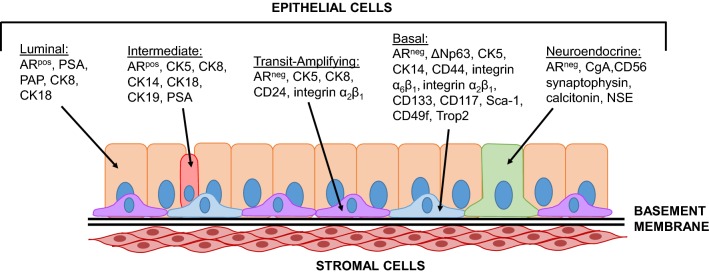
Schematic of the different cell types in the prostate and their identifying markers. The epithelial compartment is composed of three basic cell types: basal, luminal, and neuroendocrine cells, and two intermediate phenotypes. Basal cells are non-secretory cells located along the basement membrane of the epithelium and are characterized by the following markers: ΔNp63 (a member of p53 transcription factors family) [150], cytokeratins 5 and 14 (CK5 and CK14) [151, 152], CD44 [153], integrin α_2_β_1_ [65], integrin α_6_β_1_ [154, 155], CD133 [65], CD117 [66], Sca-1 [66, 156], CD49f [157], and tumor-associated calcium signal transducer 2 (Trop2) [157]. Basal cells give rise to secretory luminal cells by transitioning through intermediate states. Two intermediate phenotypes have been described: (1) transit-amplifying cells which are non-secretory and exhibit a more basal-like phenotype and (2) intermediate cells which are secretory and exhibit a more luminal-like phenotype. Both, transit-amplifying and intermediate cell types may express cytokeratin profiles similar to basal or luminal cells, however, only transit-amplifying cells have been shown to express CD24 to distinguish them from low differentiated basal cells [158] and only intermediate cells have been shown to express CK19 to distinguish them from luminal cells [159]. Luminal cells are secretory columnar cells that express high levels of androgen receptor (AR), cytokeratins 8 and 18 (CK8 and CK18), and prostatic acid phosphatase (PAP) [160]. Lastly, neuroendocrine cells are very rare cells located in the luminal layer and represent less than 1% of the prostatic epithelium. They are non-secretory, differentiated cells that express chromogranin A (CgA), CD56, synaptophysin, calcitonin, and neuron specific enolase (NSE) [161, 162]. This figure has been adapted from diagrams in related literature [163–165]

In the healthy human prostate, CD133 was first identified as a stem cell marker in a rare population (~ 1%) of basal cells that expressed α_2_β_1_ integrin (Fig. 2). This CD133^pos^/α_2_β_1_^high^ cell population was able to reconstitute prostatic-like acini with secretory activity when transplanted into male nude mice, validating their stemness and suggesting a hierarchical structure [65]. Similarly, CD133 was used in combination with other cell surface markers to identify prostate stem cells in the proximal region of mouse prostate lobes which also preferentially expressed the basal marker CK14, but not the luminal marker CK18 [66]. To validate the stemness of these cells, single CD133^pos^/Lin^neg^/Sca-1^pos^/CD44^pos^/CD117^pos^ stem cell grafts were transplanted into the renal capsule of nude mice and 14 out of 97 (~ 14.4%) of the engraftments were capable of prostate development. The above studies support the idea that CD133 expression marks a particular basal stem cell population by reflecting a hierarchically organized phenotype.

This hypothesis was supported for many years, however, more recent findings have indicated the presence of CD133 in luminal epithelial cells in both human and rodent models [67–69]. A study documented that CD133^pos^ and CD133^neg^ cells contributed equally to prostate epithelial homeostasis, bringing into question the accuracy of CD133 as a true stem cell marker [70]. Based on the evidence in the prostate alone, it appears to be clear that not all CD133^pos^ cells are stem cells and that CD133^neg^ cells may also possess stem-like properties. Several additional studies support this hypothesis by demonstrating that CD133 was expressed in differentiated epithelial cells in a variety of other organs including the pancreas [71, 72], liver [73, 74], colon [56, 75], gastric contents [76], sweat glands [77], salivary and lacrimal glands [77, 78], uterus [77], and kidneys [79]. Altogether, these studies indicate that the overall expression of human CD133 expands beyond stem cell populations and although it appears to negatively correlate with cell differentiation, it is likely not a regulator of stemness in most tissues [75]. Rather, it is more likely that CD133 is a general marker of the apical or apico-lateral membrane of the glandular epithelium [76, 77]. Furthermore, it is important to note that no stem cell population from any tissue type has been isolated to clonal purity on the basis of CD133 alone.

## CD133 as a CSC marker

CD133 has been postulated to identify CSC populations in numerous solid tumor types including several forms of brain cancer [80], prostate cancer [81], colon cancer [82], lung cancer [83], hepatocellular carcinoma [84], and ovarian cancer [85, 86], with the first 3 cancers being the most studied. In many of these studies, CD133-expressing CSCs exhibited self-renewal potential and the ability to regenerate a histologically similar tumor mass following transplantation into immunodeficient mice. Using CD133 to identify and isolate CSCs has recently become controversial for the following reasons: (1) as mentioned above it is also likely a marker of the glandular epithelium in some tissues which could make it difficult to differentiate between CSCs and non-stem like cancer cells, (2) a few studies have documented the inability of CD133^pos^ cell populations to recapitulate the original tumor morphology when xenotransplanted suggesting that CD133 may also be expressed on differentiated cells, and (3) some studies have shown CD133^neg/low^ populations are able to recapitulate the original tumor morphology as well suggesting that CD133 may not uniquely mark CSCs.

### CD133 in brain cancer

The use of CD133 to identify cancer stem cells in solid tumors was first described in pediatric tissue samples of medulloblastoma and glioma by Singh et al. [80, 87]. In these studies, more clinically aggressive brain tumor samples exhibited higher self-renewal capacity and only CD133^pos^ tissues were able to regenerate a heterogeneous tumor population in vitro, thus implying some level of hierarchy in brain tumor stem cells [87]. This data was further supported in vivo when CD133^pos^ patient brain tumor fractions were found to be tumorigenic and result in a heterogeneous population of cells wherein only approximately 20% of the cells remained CD133^pos^ following stereotactic transplantation into the frontal cortex of NOD/SCID mice [80]. Furthermore, these xenotransplantation studies revealed that as few as 100 CD133^pos^ cells were necessary to form tumors, whereas an injection of up to 100,000 CD133^neg^ cells was still insufficient to form tumors after 12 weeks in these mice.

A more recent study found that CD133^neg^ cells cultured from primary glioblastoma may be equally as tumorigenic as CD133^pos^ cells when engrafted into nude mice, but exhibited a significantly lower proliferation index (P < 0.05), potentially suggesting a prognostic value of CD133 expression in clinical specimens [88]. GeneArray analysis revealed that CD133^neg^ and CD133^pos^ samples displayed a multitude of differentially regulated genes, potentially providing some of the molecular underpinnings to describe the different growth characteristics among CD133^pos^ and CD133^neg^ brain tumors which may be exploited for tumor immunotherapy in the future. Furthermore, clinical studies have continued to support the belief that CD133 may be a significant prognostic marker regarding overall survival and progression-free survival in brain cancer patients [89–92].

### CD133 in prostate cancer

CD133 was first investigated as a prostate CSC marker using the same cell surface markers for identifying normal stem cells in the prostate. A study by Collins et al. identified prostate CSCs by isolating a population of cells which was CD44^pos^/α2β1^hi^/CD133^pos^ from 40 patient biopsies [81]. This particular cell population was postulated to be a CSC population based on its ability to self-renew, proliferate extensively, and invade in vitro. The CD44^pos^/α2β1^hi^/CD133^pos^ exhibited a self-renewal capacity that was 3.7-fold greater than the CD133^neg^ population. Additionally, CD133^pos^ cells from primary and metastatic prostate tumors showed increased proliferative potential and invasiveness compared to CD133^pos^ cells derived from benign prostate tissues. CD133 has also been used to identify CSCs in prostate cancer cell lines though the isolated CD133^pos^ cells regenerate phenotypically heterogeneous populations. For example, a CWR22Rv1 culture propagated from freshly sorted CD133^pos^ cells (> 98%) revealed that only 6.15% of cells were CD133^pos^ after 2 weeks in culture [93].

Based on the evidence of rare CD133 expression in somatic stem cells of the prostate, it was hypothesized that these CD133^pos^ CSC populations resulted from mutated normal stem cells and thus were derived from basal cells. Several studies supported this theory early on by showing that the CD133^pos^ cell populations exhibited other basal cell identifiers such as negative androgen receptor expression (AR^neg^) as shown in Fig. 2. In these studies, the CD133^pos^ cells had the ability to proliferate and differentiate into AR^pos^ cell populations, reflecting the relevance of CD133 to a hierarchically organized phenotype. However, a study by Vander Griend et al. demonstrated that CD133^pos^ sorted prostate cancer cell lines were AR^pos^ and exhibited significant growth inhibition when exposed to high-dose androgens, suggesting that these CD133^pos^ CSCs may be derived from a malignantly transformed intermediate cell rather than a normal basal stem cell [93].

Additionally, a study by Zhou et al. showed that CD133^pos^ and CD133^neg^ cell populations from immortalized primary human prostate cancer tissues demonstrated similar tumorigenicity when inoculated into NOD/SCID mice and that the CD133^neg^ cells generated significantly more prostaspheres in vitro [94]. Thus far, CD133^pos^ CSC populations have only been shown to represent roughly 1–5% of the total cell population in prostate cancer cell lines [93]. However, it has been suggested that CD133^pos^ populations could be enriched in vitro through chemotherapy or radiotherapy, postulating that these cells exhibit at least some level of chemo/radioresistance [95]. A study evaluating the circulating tumor cells from 12 metastatic castration-resistant prostate cancer patients established that the CD133^pos^ cells exhibited higher proliferative potential than their CD133^neg^ counterparts in 10/12 patients [96], suggesting that CD133^pos^ cells do have enhanced potential for cell division despite chemo/radiotherapy. Due to the inconsistent evidence supporting CD133 as a prostate CSC marker, it is still unclear whether CD133 plays a direct role in prostate CSC maintenance or if it is simply correlated to more aggressive disease.

### CD133 in colorectal cancer

In 2007, two separate studies found that a small population of CD133^pos^ colon cancer cells were able to generate a tumor when transplanted subcutaneously and into the renal capsule of immunodeficient mice, whereas, CD133^neg^ colon cancer cells could not [82, 97]. In both studies, the tumors were morphologically similar to the original tumor and were capable of re-establishing tumor heterogeneity. In contrast, Shmelkov et al. demonstrated that CD133^pos^ and CD133^neg^ patient metastatic tumor cells formed colonospheres in in vitro cultures and were serially tumorigenic in a NOD/SCID mice [56]. Furthermore, the CD133^neg^ cells formed more aggressive tumors and expressed other typical phenotypic markers of CSCs, including CD44, whereas the CD133^pos^ fraction was composed of primarily CD44^low^ cells. Using CD133 as a colon cancer stem cell marker has continued to be controversial as some studies have found other markers, such as CD44, EpCAM (CD326), and CD166, to be much more robust at identifying colorectal CSCs [98, 99]. Numerous recent clinical studies, however, have indicated that CD133 exhibits a significant prognostic value for predicting patient survival in colorectal cancer [100–103].

### CD133 in lung cancer

CD133 has been implicated as a CSC marker in both non-small cell lung carcinomas and small cell lung carcinomas [104]. A study by Eramo et al. showed that lung cancer CD133^pos^ cells were able to grow as tumor spheres indefinitely [83]. Similarly, subcutaneous injection of 10^4^ CD133^pos^ lung cancer cells in SCID mice readily generated tumor xenografts phenotypically identical to the original tumor, whereas a tenfold higher number of CD133^neg^ cells were not tumorigenic in the same mice. Upon differentiation, CD133^pos^ lung cancer cells acquired specific lineage markers, lost their tumorigenic potential, and lost their CD133 expression, indicating their stem-like potential. However, a more recent study showed that CD133 was not a robust marker for identifying CSCs in non-small cell lung carcinoma [105]. A study by Zhang et al. demonstrated that CD133^pos^ and CD133^neg^ cell populations showed similar tumorigenic potential in mice. While both studies used the same CD133-targeted antibody for cell separation, the authors attribute their different results to the use of a more sensitive mouse xenotransplantation model (NOD-SCID IL2rγ^−/−^), suggesting that the wide spectrum of results regarding CD133 as a CSC marker can be dramatically different based on the different types of assays used in each study.

### CD133 in liver cancer

A number of studies have documented CD133 as a CSC marker in liver cancer. A study in 2006 showed that CD133^pos^ Huh-7 hepatocellular carcinoma cells exhibited higher proliferative potential in vitro and greater tumorigenic capacity in SCID mice compared to CD133^neg^ cells [84]. Along with the CSC hypothesis, multiple studies have also shown that CD133^pos^ cell populations in liver cancer confer chemo- and radio-resistance in liver cancer [106–108]. Numerous molecular mechanisms have been investigated to better understand how CD133^pos^ liver CSCs evade conventional therapies including resistance to interferon-gamma-induced autophagy [106] and preferential activation of protein kinase B (Akt/PKB) and B-cell lymphoma-2 (Bcl-2) cell survival response [107]. Similarly, Ding et al. demonstrated that CD133^pos^ liver cancer cells were resistant to transforming growth factor β (TGF-β)-induced apoptosis and this effect could be blunted using a mitogen-activated protein kinase 1 (MEK1) inhibitor [109]. Interestingly, a recent study indicated that the location of CD133 on the CSC may play an important role on the aggressiveness of the cancer and the prognosis of the liver cancer patient [110]. This study analyzed cancerous tissues and pair-matched adjacent normal liver tissues from 119 hepatocellular carcinoma patients and revealed that cytoplasmic CD133 expression was correlated with poor prognosis, while nuclear CD133 expression was correlated with favorable prognosis.

### CD133 in ovarian cancer

CD133, in combination with other cell surface markers has been shown to identify CSC populations in ovarian cancer as well. A study by Cioffi et al. revealed that ovarian cancer cell lines that co-expressed CD133 and CXCR4 exhibited stem cell properties that at least partially regulated tumor development, migration, and chemoresistance [86]. Research by Choi et al. demonstrated that Only ALDH^pos^/CD133^pos^ ovarian cancer cell population could produce progeny with varying ALDH and CD133 statuses, suggesting that these cells can differentiate to form a heterogenous population of cancer cells [111]. This study also found that bone morphologenetic protein 2 (BMP2) promoted the expansion of the ALDH^pos^/CD133^pos^ CSC populations in vitro and thus attributed to increased tumor growth and chemoresistance when assessed in vivo. A recent study also showed that CD133 expression plays a role in cell homing during metastasis by increasing cell adhesion in the peritoneal tissue in models of ovarian cancer [112]. These results suggest that targeting CD133 may lead to improved therapies which will reduce the risk of tumor recurrence by minimizing invasion and metastatic potential.

### CD133 in other cancers

While CD133 is less investigated in other cancers, it has been briefly documented in other solid tumor types. Studies in breast cancer have indicated that CD133^pos^ cells display heightened tumorigenicity, self-renewal in vivo, increased metastatic potential, and the capacity to give rise to functional and molecular heterogeneous cell populations [113, 114]. In stark contrast to most other cancers, overexpression of CD133 has been associated with non-metastatic disease and longer survival in renal carcinoma patients, whereas low expression of CD133 was considered to be a predictor of poor disease prognosis [115, 116]. In pancreatic cancer, one study showed that a distinct population of CD133^pos^/CXCR4^pos^ expressing CSCs were exclusively tumorigenic, mediated metastasis, and highly resistant to chemotherapy [117], however, a more recent study countered this notion by suggesting that CD133 is not a robust marker for CSCs in pancreatic cancer [118]. Similarly, two studies recently indicated that CD133 does not identify CSC populations in gastric cancer cell lines or primary human gastric tumors [119, 120]. CD133 has also been used to identify CSCs in bone tumors [121, 122]. While the evidence of its role in stem cell maintenance has yet to be elucidated, multiple studies have concluded that CD133^pos^ cells in bone tumors exhibit higher quantities of stemness related transcription factors, such as NANOG, MYC, OCT 3/4, and SOX2, than CD133^neg^ populations [121, 123].

## Clinical significance of CD133 in cancer

Overall, the evidence regarding the accuracy of using CD133 as a CSC antigen is still controversial. Based on the above evidence, the results vary significantly on a number of factors, including experimental design, culture conditions, cancer subtype, mouse species, cell line variability, tumor microenvironment, etc. While CD133 may not be exclusive to CSCs, there does appear to be a correlation among CD133 expression and CSC enrichment in most of these studies. It is implied that CSCs are highly plastic cells, thus a better understanding of the molecular foundation of CD133 regulation is needed to fully understand its functional role in CSC maintenance and cancer progression.

Despite the lack of knowledge regarding the molecular underpinnings of CD133 in cancer, a majority of the current studies do suggest that CD133 exhibits a significant prognostic and predictive value to overall survival, disease-free survival, and progression-free survival in many different solid cancers [31]. In two comparative gene expression profiling studies, CD133 expression correlated with predicting glioblastoma patient outcomes and response to therapy [124, 125]. Given that both CSCs and CD133^pos^ cell fractions have been shown to exhibit chemo- and radio-resistance [83, 117, 126–129], the ability to predict how patients will respond to therapy could fulfil a significant unmet clinical need in many cancers where CD133 is overexpressed.

## Limitations of the clinical significance of CD133 in cancer

Investigating the role of CD133 plays in cancer has relied on using immunohistochemical methods to detect protein expression and flow cytometry for sorting CD133^pos^ cells. A major limitation to both of these approaches is that they require the use of antibodies for the accurate identification of CD133-expressing cells. Since CD133 is a glycoprotein with multiple N-glycan structures, it is highly sensitive to glycosylation modification, which may influence antibody binding. To date, most studies use one of the commercialized Miltenyi antibody clones, CD133/1 (AC133 or W6B3C1) or CD133/2 (AC141 or 293C3), which bind to two different, glycosylated epitopes on the EC3 region of CD133 (Fig. 1). Studies have suggested that these epitopes may become inaccessible due to alternative splicing or be masked as a result of differential glycosylation [27, 130]. Additionally, these epitopes are poorly defined and cross-reactivity may occur with other glycosylated epitopes, yielding inaccurate results [29]. In all scenarios, the epitopes become unavailable for accurate detection, potentially validating many of the inaccuracies with CD133 expression in the literature. A few other antibodies have been developed for non-glycosylated regions of the CD133 protein, however, they have been even less successful in detecting CD133 in various assays [131]. Similarly, most antibodies are validated in mouse models, thus the low level of amino acid conservation across species may also explain the lack of cross-species immunoreactivity when assayed on human tissues [28].

## Therapeutic strategies targeting CD133

Despite the contradictory data regarding the use of CD133 to identify CSCs, it has been consistently reported that high levels of CD133 expression correlate with shorter patient lifespan and more aggressive disease. Many of the therapeutic strategies targeting CD133 have focused on using the overexpression of CD133 for targeted drug delivery. Unfortunately, because the currently available antibodies are limited in their ability to detect CD133 splice variants and aberrantly post-translationally modified CD133, there has been very little progress in therapeutic development.

### Immunotoxins

In vitro studies using the anti-CD133 monoclonal antibody (mAb), AC133, conjugated to a genetically modified cytolethal distending toxin (^C178A^BC-CD133Mab) were able to inhibit the proliferation of CD133^pos^ head and neck squamous cell carcinoma cells by causing significant DNA damage and subsequent growth arrest [132]. For reasons unknown, this drug never made it to phase I clinical trials. In 2010, Swaminathan et al. developed a novel anti-human CD133 mAb, termed clone 7, which recognizes an unglycosylated extracellular domain of CD133 [133]. Future studies developed a deimmunized anti-CD133 targeted toxin by conjugating this novel mAb to a mutated pseudomonas endotoxin (dCD133KDEL) [134]. The resulting fusion protein, dCD133KDEL, selectively inhibited the growth of two head and neck squamous cell carcinomas and did not inhibit the viability of hematopoetitic lineages, suggesting its significant promise as an anti-cancer agent. Additionally, CD133^pos^ squamous carcinoma cells that were pretreated with dCD133KDEL prior to xenotransplantation exhibited less tumorigenicity than those which had not been pretreated with dCD133KDEL. Furthermore, tumors treated with multiple intratumoral injections of dCD133KDEL showed marked growth inhibition leading to complete degradation of the tumors.

To expand the binding capacity of dCD133KDEL to a broader range of CSCs, Waldron et al. conjugated an additional anti-EpCAM scFv converting it to a deimmunized bispecific targeted toxin (dEpCAMCD133KDEL) [135]. This bispecific targeted toxin potently inhibited protein translation and proliferation in breast and colon carcinoma cell lines. Finally, dEpCAMCD133KDEL also caused tumor regression in an in vivo model of head and neck squamous cell carcinoma. When compared to the single targeted tumor toxin, the bispecific tumor toxin exhibited greater tumor growth inhibition.

### T-Cell therapy

Zhao et al. demonstrated that arming activated T-cells (ATCs) with a bispecific antibody for AC133 and CD3, termed MS133, could produce anti-tumor effects in vitro and in vivo [136]. Upon treatment with the MS133 armed ATCs, cytotoxicity of CD133^pos^ colorectal tumor cells was observed. Likewise, MS133 armed ATCs displayed significant tumor growth retardation in a subcutaneous colorectal xenograft model with NOD/SCID mice, as well as no obvious change in body weight indicating that this treatment strategy displays very little toxicity in the mice.

A phase I clinical trial recently demonstrated the utility of chimeric antigen receptor-modified T-cell (CART) directed CD133 therapy in patients with hepatocellular carcinoma, pancreatic carcinomas, and colorectal carcinomas [137]. While only 3 out of the 23 patients achieved partial disease remission, 14 patients remained stable, and 21 patients did not develop any additional detectable metastatic lesions during the study. In general, majority of the experienced minimal adverse effects, however, hyperbilirubinemia was observed in 3 of the patients which should be taken into consideration if the patient is susceptible to biliary obstruction.

### Natural killer cell therapy

A series of natural killer cell (NK) therapies targeting CD133 have also been developed. The first was a novel bispecific killer cell engager (BiKE) capable of targeting CSCs by combining a gene encoding a human anti-CD16 scFv to an anti-CD133 scFv [138]. The CD133 component allows the BiKE to recognize CSCs and the CD16 component allows the NK cells to recognize the BiKE promoting antibody-dependent cell mediated cytotoxicity. The BiKE greatly enhanced the NK-cell killing of human CD133-expressing Caco-2 colorectal carcinoma cells as indicated by in vitro chromium release cytotoxicity assays. Similarly, a trispecific NK cell engager (TriKE) comprising single-chain variable fragments (scFvs) binding CD16 on NK cells, CD133 on CSCs, and a modified IL-15 crosslinker to enhance NK cell response was also developed [139]. The IL-15 crosslinker significantly improved the anti-cancer effects of the TriKE by inducing cytotoxic degranulation. A tetraspecific killer engager (TetraKE) consisting of scFvs for binding to CD16, EpCAM, and CD133, with an IL-15 crosslinker was also developed [140]. The TetraKE exhibited improved activity, induction of proliferation, and prolongation of survival of NK cell effectors, as well as, increased NK-cell performance when compared to other individual antibodies and BiKEs. These data suggest that NK-mediated therapies may be a promising strategy for immune targeted annihilation of cancer cells.

### Antibody conjugated nanoparticles

In 2013, a study used the anti-CD133 mAB, AC141, conjugated to nanoparticles loaded with paclitaxel, a frequently used anti-cancer agent, to inhibit tumor progression in an orthotopic mouse model of breast cancer [141]. The CD133-targeted nanoparticles (CD133NPs) were capable of internalization into CD133-expressing cell lines and reduction of the CSC population. In vivo studies revealed that the CD133NPs exhibited a 70% regression in tumor volume, whereas, the free paclitaxel and IgG-nanoparticle control only led to a 33 and 43% reduction in tumor volume, respectively. Although the tumors relapsed in all treatment groups, the CD133NPs were significantly more effective at slowing the growth of recurrent tumors. Shortly after, a commercial anti-CD133 polyclonal antibody conjugated to paclitaxel loaded nanoparticles exhibited anti-tumor effects in liver cancer in vitro and in vivo models [142]. The targeted nanoparticles inhibited the rate of tumor formation by 69.3% compared to the non-targeted nanoparticles which only inhibited tumor formation by 57.4%. Additionally, mice that received targeted nanoparticle therapy lived 21 days longer on average than those which received non-targeted therapy.

### Aptamers

Studies have also used CD133-targeted aptamers for nanoparticle delivery. Aptamers are small single-stranded RNA or DNA oligonucleotides (~ 20–60 nucleotides) that differ from antibodies in that they are non-immunogenic nor toxic and chemical synthesis of the aptamers allows for a significant reduction in lot to lot variability during bioproduction [143]. One study used a PEGylated nanoparticle conjugated with a CD133-targeted RNA aptamer (Apt-PEG-AcCMC-SN38) to deliver a poorly soluble chemotherapeutic, SN38, to CD133-expressing colorectal cancer cells [144]. In this study, CD133^pos^ cells exhibited significant growth inhibition when treated with Apt-PEG-AcCMC-SN38, while the viability of CD133^neg^ cell lines remained unaffected.

Similarly, two other CD133-targeted RNA aptamers (CD133-A15 and CD133-B19) have been developed and tested for their anti-cancer effects in vitro [145]. Both CD133-targeted aptamers demonstrated comparable selectivity in CD133^pos^ cell lines when compared to the AC133 antibody. Furthermore, both aptamers demonstrated superior tumor penetration from 30 min to 4 h following treatment with retention lasting up to 24 h in an HT-29 colon cancer 3-D tumor sphere model, suggesting its utility as a therapeutic while minimizing undesirable adverse effects.

### Other emerging therapies

Immunocellular therapeutics has developed a CD133-targeted dendritic-cell based immunotherapy, termed ICT-121, to treat patients with recurrent glioblastoma. Given the recurrent nature of this disease, these patients are often resistant to other chemotherapeutic and radiotherapeutic agents. ICT-121 was generated by collecting autologous monocytes and allowing them to mature into dendritic cells in the laboratory. Once mature, these cells were loaded purified peptides from the CD133 antigen [146]. An ongoing phase I clinical trial (NCT02049489) is still investigating the safety and effectiveness of ICT-121 administration to recurrent glioblastoma patients, however, preliminary results suggest that ICT-121 is safe and well tolerated and an effective immune response is currently being observed in a subset of patients [147].

Radioimmunotherapy approaches targeting CD133 are also being investigated. A study by Weng et al. radiolabeled an AC133 mAb with iodine-131 (^131^I) and delivered it to nude mice bearing colon cancer xenografts [148]. Both, tumor volume doubling time and overall survival time were increased in the ^131^I-AC133 compared to the ^131^I-IgG control or the AC133 antibody alone, suggesting its potential as an anticancer agent.

A novel approach using near-infrared photo immunotherapy (NIR-PIT) targeting CD133 in glioblastoma is also proving to be an effective option for efficient eradication of CSCs [149]. In this study, an AC133 mAb was conjugated to an IR700 photoabsorber dye. When activated by NIR light, the photoabsorber becomes activated and causes the cell to swell and ultimately leads to necrosis. Subcutaneous and orthotopic mouse models with CD133^pos^ glioblastoma stem cells revealed that the NIR-PIT-AC133 mAb was able to shrink both tumor models and extend the lifespan of these mice.

## Conclusions

While much progress has been made in recent years to better understand the predictive and prognostic power of CD133 in solid cancers, the utility of CD133 to mark CSCs is still very controversial. One of the reasons for this is that the experiments used to test the presence of CD133 on normal and cancer cells are not congruent. Different cell culture conditions, animal models, and assays to determine cell viability, proliferation, and self-renewal, are yielding extremely conflicting results. Furthermore, current methods for the accurate detection of CD133^pos^ cells are less than optimal. Most studies are currently using the commercialized antibodies, AC133 or AC141, which are only able to detect glycosylated CD133 on the EC3 domain. Developing new molecules which can detect CD133 splice variants and post-translationally modified CD133 could significantly improve the accuracy of these experiments and lead to more comparable results. Additionally, these improved molecules could provide theranostic benefits, considering CD133 overexpression has been correlated with poor prognosis and reduced overall survival in a number of different cancers.

## Abbreviations

CRC: cancer stem cell
EC1: extracellular domain 1
IC1: intracellular domain 1
IC2: intracellular domain 2
EC2: extracellular domain 2
EC3: extracellular domain 3
IC3: intracellular domain 3
PROM 1: prominin 1
HIF-1α: hypoxia inducible factor-1α
HSC: hematopoietic stem cell
NSC: neural stem cell
FACS: fluorescence-activated cell sorting
mAb: monoclonal antibody
AR: androgen receptor
Bcl-2: B-cell lymphoma-2
TGF-β: transforming growth factor β
MEK1: mitogen-activated protein kinase kinase 1
BMP2: bone morphologenetic protein 2
ATC: activated T cell
CART: chimeric antigen receptor-modified T-cell
BiKE: bispecific killer cell engager
TriKE: trispecific NK cell engager
TetraKE: tetraspecific killer engager
CD133NP: CD133-targeted nanoparticles
^131^I: iodine-131
NIR-PIT: near-infrared photoimmunotherapy
